# Supplementary material for: Control of the temporal and polarization response of a multimode fiber
Source: Nat Commun. 2019 Nov 8;10:5085. doi: 10.1038/s41467-019-13059-8 (PMC6841946; doi:10.1038/s41467-019-13059-8)
Supplement: Supplementary file 1 — Supplementary Information [file 41467_2019_13059_MOESM1_ESM.pdf]

Supplementary Material for "Control of the temporal and polarization response of a multimode fiber"

Mickael Mounaix[1,*] and Joel Carpenter[1]

[1]*School of Information Technology and Electrical Engineering, The University of Queensland, Brisbane QLD 4072, Australia*

This document provides supplementary information to “Control of the temporal and polarization response of a multimode fiber”. It provides additional details on the experimental setup, the temporal correlation of the different Time Resolved Transmission Matrices, extra data processing of the maximum eigenstate at different delays, and additional plots on the multiple delay control.

* m.mounaix@uq.edu.au

Supplementary Note 1. ADDITIONAL INFORMATION ON THE EXPERIMENTAL MEASUREMENT OF THE MULTI SPECTRAL TRANSMISSION MATRIX

The experimental setup presented in Figure 1 enables us to shape the input Gaussian beam from the laser into an arbitrary spatial and polarization state. A polarization controller (not shown in Figure 1) aligns the input polarization state such that the power is split equally between the H and V ports of the first polarization beam splitter. The vertical polarization beam is rotated with an half waveplate, as required for the LCOS-SLM beam shaping. These two beams are then independently manipulated on the left and the right sides of the spatial light modulator [1]. For clarity purposes, on Figure 1 the SLM is illustrated as transmissive, however in reality the SLM is used in reflection. The two polarization beams are then recombined using polarization optics and imaged onto the input facet of the multimode fiber.

In order to achieve amplitude and phase modulation of the input beam with a phase-only SLM, spatial filtering in the Fourier plane of the SLM is required. This spatial filtering is performed by the multimode fiber itself, which is located in the first diffractive order of the SLM in the Fourier plane.

The transmission matrix is measured in the mode basis of the multimode fiber. The $N_{\text{input}}/2$ modes per polarization are sent one after another onto the input facet of the multimode fiber.The input modes can be numerically calculated based on the core dimension, the refractive index properties of the fiber and the waist of the beam. For a given input mode (for either the horizontal or the vertical polarization), the phase pattern to be displayed onto the SLM is calculated with the Gerchberg-Saxton algorithm [2]. After propagation through the multimode fiber, the transmitted output field is then measured with the digital off-axis holography setup simultaneously for the two polarization states. The output field is overlapped with the $N_{\text{output}}/2$ modes per polarization. We decompose the output field in the LG basis for computation complexity reasons. We are decomposing the output field onto a full basis, therefore the choice of the basis will not alter the results. The decomposition is made with many more modes until almost all power is accounted for, which explains why we have $N_{\text{output}} > N_{\text{input}}$. The mode decomposition at the output, for a specific input mode, forms a column of the transmission matrix.

The Multi Spectral Transmission Matrix $U(\lambda)$ is the stack of transmission matrices for all the measured spectral components. Supplementary Figure 1a shows the amplitude of three transmission matrices measured at different wavelengths. Supplementary Figure 1a3 shows a zoom of the amplitude of transmission matrix at $\lambda = 1510$ nm. For a given input mode, the polarization content of the output field is interlaced, which differs slightly with the way $U(\lambda)$ is written in Equation 4. Nonetheless, one can re-organize the mode decomposition coefficients within the transmission matrix to match Equation 4.

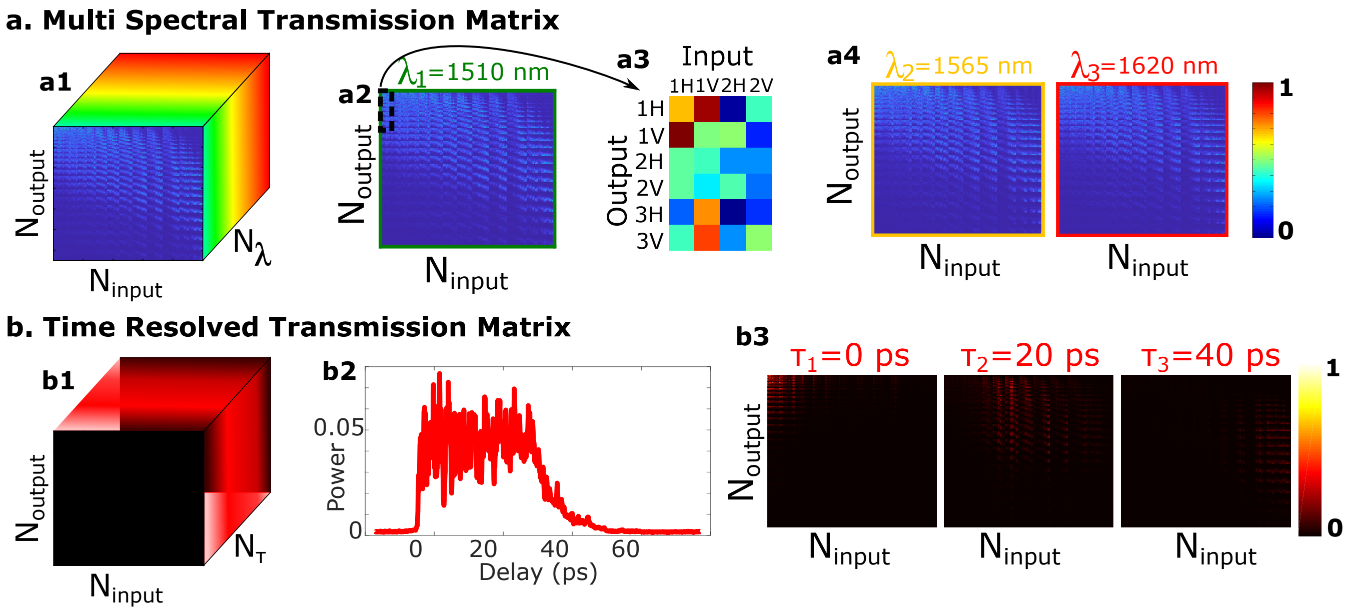

Supplementary Figure 1. **(a)** Multi Spectral Transmission matrix of the multimode fiber. (a1) Artistic representation of the experimentally measured MSTM. Its dimensions are $N_{\text{input}} = 254$ input modes, $N_{\text{output}} = 650$ output modes, and $N_{\lambda} = 1273$ spectral components. (a2) Amplitude of the transmission matrix measured at $\lambda_1 = 1510$ nm. (a3) Zoom of (a2). The polarization information is interlaced both at the input and at the output. ij stands for mode i, i is an integer from 1 to $N_{\text{input}}/2$, on the polarization state j, j being either H (for horizontal) or V (for vertical). (a4) Amplitude of the transmission matrices measured at $\lambda_2 = 1565$ nm and $\lambda_3 = 1610$ nm. **(b)** Time Resolved Transmission matrix of the multimode fiber. (b1) Artistic representation of the TRTM, calculated with a Fourier transform of the experimentally measured MSTM along the spectral axis. (b2) Total power of the transmission matrix per delay time. (b3) Amplitude of the transmission matrices at different delay times: $\tau_1 = 0$ ps, $\tau_2 = 20$ ps and $\tau_3 = 40$ ps.

Supplementary Note 2. MODE COUPLING IN THE MULTIMODE FIBER

The question of "how strong is the mode coupling?" is a natural question to ask, however extracting the answer experimentally has a lot of nuance to it. In simulation the coupling strength if something you enter numerically, in experiment you're not privy to what's going on along the length of the fiber, only what you observe at the ends. Importantly, you also have to distinguish between genuine mode coupling inside the fiber, and "pretend" mode coupling, which is a result of your choice of measurement basis in the experiment. This topic has been discussed in [3]. In fact in a typical fiber, there's the dynamics of multiple strengths of mode coupling all occurring simultaneously between different groups of modes. We show in Supplementary Figure 1a2 the transmission matrix at 1565nm. The matrix is in the linearly polarized mode basis, both at the input and at the output. The matrix is not diagonal, but not completely filled either. There is no universal metric to describe mode coupling, particularly in the regime where modes are not completely coupled. We've chosen to use here the mode participation number MP per input mode based on the mode decomposition at the output, which quantifies how light is distributed over the different modes [4].

$$\text{MP} = \frac{(\sum_{i=1}^{N_{\text{output}}} |t_i|^2)^2}{N_{\text{output}} \times \sum_{i=1}^{N_{\text{output}}} |t_i|^4} \tag{1}$$

With N_{output} the number of modes at the output and t_i the coefficients of the mode decomposition of the $i-th$ mode at the output.

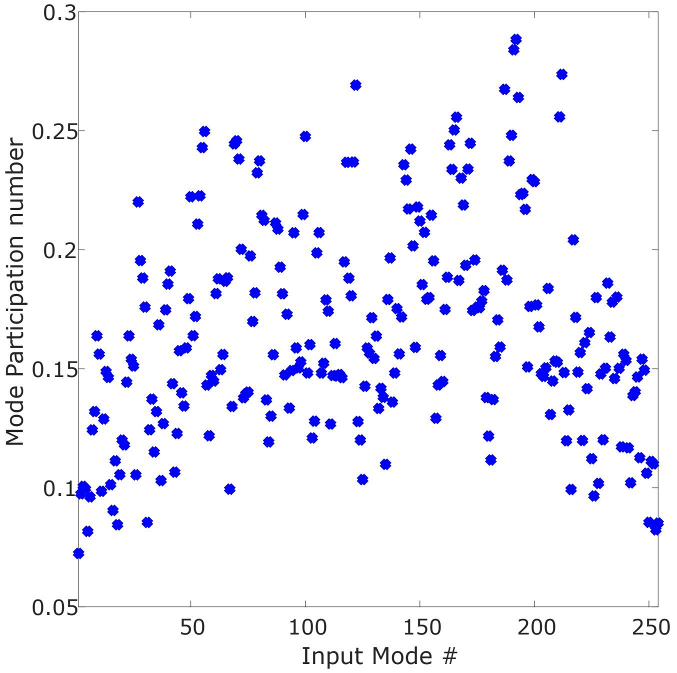

Supplementary Figure 2. Mode participation number at 1565 nm for every input mode.

Supplementary Figure 2 shows MP for the different input modes. An isotropic TM would have a MP>0.5. The average over all input modes is 0.16, which means we are in a mode coupling regime that is not strong [4].

Supplementary Note 3. CORRELATION BETWEEN TIME RESOLVED TRANSMISSION MATRICES

The Time Resolved Transmission matrix is calculated with the experimentally measured MSTM, with a Fourier transform along the spectral axis. Supplementary Figure 1b2 illustrates the total power per delay. Supplementary Figure 1b3 shows the transmission matrix at three different delay times: 0 ps, 20 ps and 40 ps. The low order modes tend to have most of their power at early arrival times, while the higher order modes have most of their power at longer arrival times.

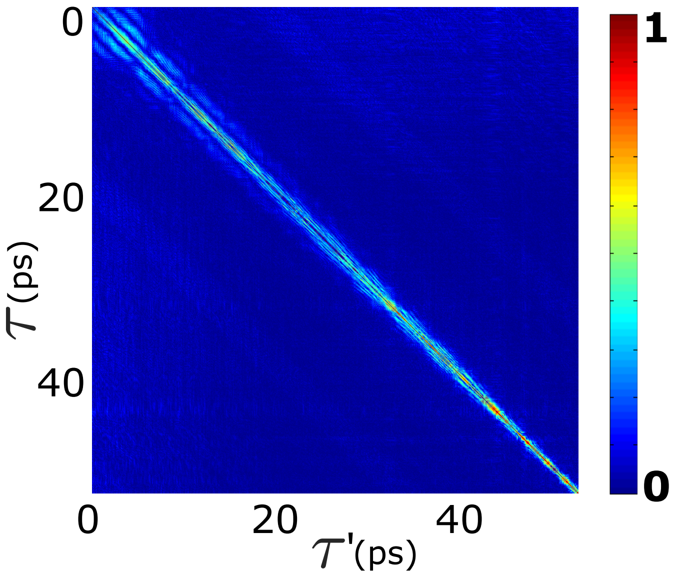

Supplementary Figure 3. 2D representation of the temporal correlation C(τ,τ') between time resolved transmission matrices at delay τ and τ', for a set of τ and τ' varying between 0 ps and 50 ps.

In a step-index multimode fiber, the impulse response of each mode is broad in the time domain, as each input mode is coupled along propagation with other modes that have different group delays. When we manipulate the impulse response at different delay times, the impulse response could interfere if the delay times are close to each other. We can quantify this minimum time interval where the transmission matrices won't be correlated to each other with the temporal correlation C(τ,τ'). We define the correlation C(τ,τ') as the overlap between the transmission matrix U(τ) at delay τ and the transmission matrix U(τ') at delay τ':

$$C(\tau, \tau') = \left| \frac{\sum_{i=1}^{N_{\text{input}}} \sum_{j=1}^{N_{\text{output}}} U_{ij}(\tau) U_{ij}^*(\tau')}{\sqrt{\sum_i \sum_j |U_{ij}(\tau)|^2} \sqrt{\sum_i \sum_j |U_{ij}(\tau')|^2}} \right| \tag{2}$$

with $U_{ij}(\tau)$ the coefficient of the transmission matrix at delay τ relating the $i - th$ input mode and the $j - th$ output mode, and * the complex conjugate operator. With this definition, we thus have C(τ,τ)=1, which corresponds to the auto-correlation of the time resolved transmission matrix at time τ.

Supplementary Figure 3 shows the 2D map of C(τ,τ') for τ and τ' between 0 ps and 50 ps. On average, for a given delay τ, the correlation C(τ,$\tau + \delta\tau$) is negligible (e.g C(τ,$\tau + \delta\tau$)<0.1) for $\delta\tau \sim 1 - 2$ ps.

Supplementary Note 4. EXTRA DATA PROCESSING OF THE MAXIMUM EIGENSTATE AT DIFFERENT DELAY TIMES

We provide in this section extra processing of the maximum eigenstates that are presented in Figure 2 of the main manuscript.

Supplementary Note 4.A. Mode decomposition at the output at central wavelength as a function of delay

We provide an additional processing of the data of Figure 2b of the main manuscript. We plot in Supplementary Figure 4 the mode decomposition at the central wavelength (1565 nm) of the output field of the different eigenmodes excited in Fig.2b, for different delays. We show in Supplementary Figure 4a the amplitude of the mode decomposition as a function of arrival time, and in Supplementary Figure 4b we plot the center of mass of the mode decomposition per delay time. Intuitively, the earlier modes corresponds to lower order modes (that have a higher group velocity and thus getting out of the fiber faster than the higher order modes), and the later modes are a composition of higher order modes (that have a lower group velocity). For mid-delay, the mode mixing is more complex and there is a combination of low order modes and higher order modes according to Supplementary Figure 4a.

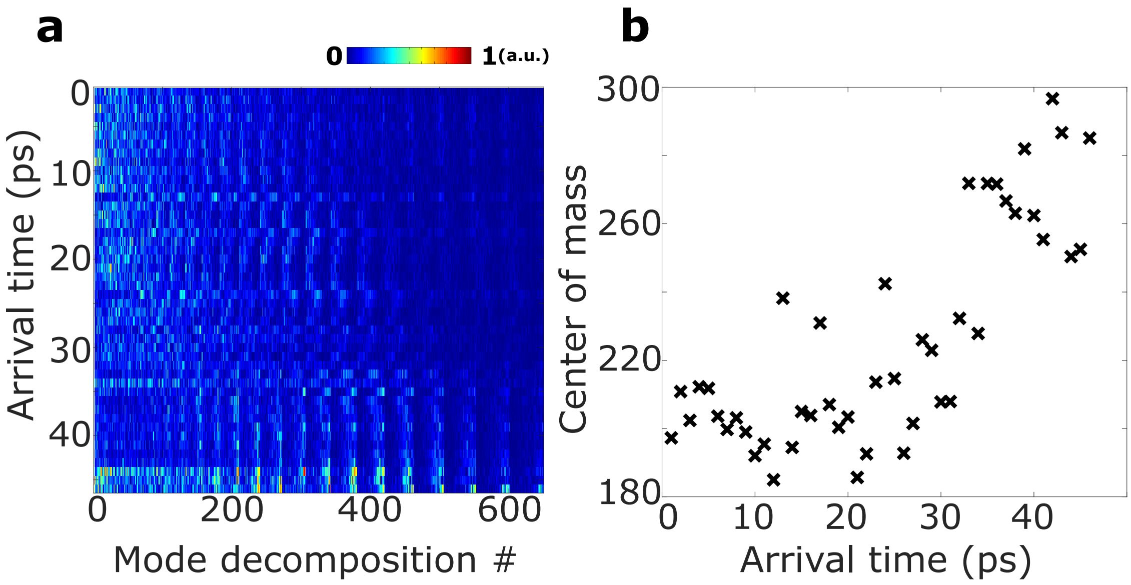

Supplementary Figure 4. Analysis of the mode decomposition of the output field at the central wavelength (1565 nm) for the input eigenmodes enhancing transmission at different delays (same dataset as Fig.2b of the main manuscript). (a) The amplitude of the output mode decomposition is plotted for the different arrival times. (b) Center of mass of the output mode decomposition as a function of arrival time.

Supplementary Note 4.B. Mode Participation number for the max eigenstate at each delay

In Supplementary Figure 5a, we plot the mode participation as defined in Eq. 1 for each eigenstate maximizing the impulse response at a chosen delay. As expected, the number of modes at early and late delays is smaller than at mid delays, as demonstrated with principal modes [4].

Supplementary Note 4.C. Impulse responses of the maximum eigenstate per delay and field correlation in the temporal domain

In Supplementary Figure 5b, we plot in a 2D graph the individual impulse response of the first maximum eigenstate at each delay time, as an extension to the plot of Fig. 2a of the main document. The x-axis corresponds to the delay axis and the y-axis corresponds to each individual impulse response arriving at different delay times between 0 ps and 45 ps.

To estimate the time width of each individual impulse response, we calculate the square of the field correlation $|F_E(\Delta\tau)|^2$ in time [5], with:

$$F_E(\Delta\tau) = \frac{E(\tau_{\text{max}})E^*(\tau_{\text{max}} + \Delta\tau)}{\Big(I(\tau_{\text{max}})I(\tau_{\text{max}} + \Delta\tau)\Big)^{1/2}} \tag{3}$$

With $E(\tau_{\text{max}})$ the reconstructed field constituted of the mode decomposition at delay τ_{max}, corresponding to the chosen enhanced delay. We plot $|F_E(\Delta\tau)|^2$ for all the impulse responses from Supplementary Figure 5b in Supplementary Figure 5c. At late delays, the temporal profiles of the achieved eigenstates are more sharply peaked compared to mid delays. This could be explained by the mode participation number and the lower mode coupling at these delays, as the modes are near cut-off [1, 3].

Supplementary Note 4.D. Spatial reconstruction intensity image at each delay

Finally, we provide the spatial reconstruction at each delay for both polarization states in Supplementary Figure 6, as an extension to the inset of Fig. 2a. The modes at late delays are using the full aperture of the fiber and correspond to higher order modes that look like "textbook" eigenmodes, while the modes at early delays are mostly low order modes.

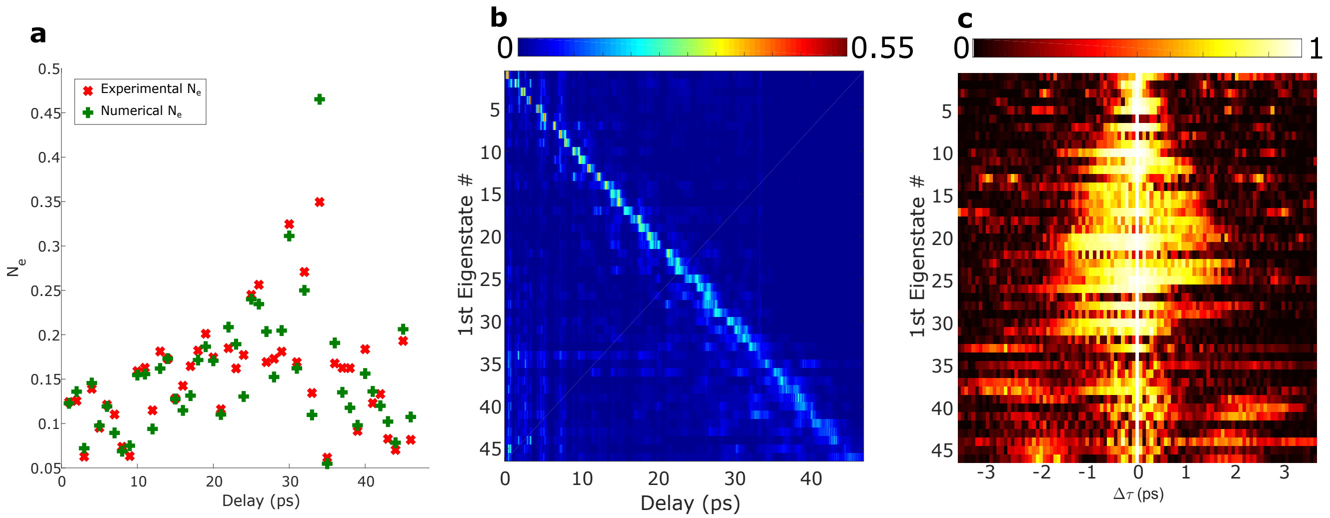

Supplementary Figure 5. Analysis of the mode decomposition of the output field at the central wavelength (1565 nm) for the input eigenmodes enhancing transmission at different delays (same dataset as Fig.2b of the main manuscript). (a) The amplitude of the output mode decomposition is plotted for the different arrival times. (b) Center of mass of the output mode decomposition as a function of arrival time.

Supplementary Note 4.E. Simulation with an input pulse of given duration

In our experiment we are not sending a pulse. Instead, we are using swept wavelength holography to retrieve the spectral field response. We provide here some simulation results, based on the experimentally measured MTM, of the impulse response we would measure with an input short pulse. We then calculate the enhancement ratio, as well as the field correlation in the temporal domain. A Gaussian short pulse in the time domain of given duration corresponds to a Gaussian amplitude distribution in the spectrum with a flat spectral phase. We thus filter the spectral amplitude of our experimentally measured MTM with such Gaussian amplitude along the wavelength axis. We numerically propagate the input field with this spectrally filtered MTM and get the spectrally resolved output field. A Fourier transform along the frequency axis gives the time resolved output field upon numerical propagation of the input pulse, from which we get the impulse response. We can then extract the enhancement factor as a function of delay time for different input pulse durations, as well as the field correlation.

We plot the impulse response of the maximum eigenstate at a single delay, for instance $\tau_{\max}$=14 ps, for either an input pulse of duration 260 fs (whose spectral Bandwidth $\simeq$ 20% of our full spectral measurement) or 1.3 ps (whose spectral Bandwidth $\simeq$ 3% of our full spectral measurement) in Supplementary Figure 7a and in Supplementary Figure 7d. The temporal features of the background outside $\tau_{\max}$, as well as the width of the peak at $\tau_{\max}$ have been averaged out compared to Fig. 2a, as they have now an average width that is the input pulse duration. To confirm this, we plot the field correlation in Supplementary Figure 7b and Supplementary Figure 7e for both input pulse durations. In contrast with Supplementary Figure 5c, the time width of the peak of the impulse response seems constant for the different delays and identical to the input pulse duration, as studied in [5] for spatio-temporal focusing in a single position of an input Gaussian pulse. In Supplementary Figure 7c and Supplementary Figure 7f we plot the enhancement ratio for the two different input pulse durations. There is no clear trend for enhancement ratio as a function of the input pulse width for our MMF in this mode coupling regime. As mentioned above, there are dynamics of both strong coupling and weak coupling occurring amongst different groups of modes/delays in the fibre, and it can be quite sensitive to very specific details of the fiber parameters.

Supplementary Note 5. INCOMPLETE CONTROL OF THE POLARIZATION

We provide an additional numerical result of the importance of polarization control. Let's assume firstly that we can control both polarization at the input (on the SLM). We choose the same τ as Fig.4 of the main document, that is $\tau = 8.5$ ps. We can then control the full transmission matrix $U(\tau)$

$$U(\tau) = \begin{bmatrix} U_{HH}(\tau) & U_{HV}(\tau) \\ U_{VH}(\tau) & U_{VV}(\tau) \end{bmatrix} \tag{4}$$

If we want to enhance only the H state at the output, then we can use only $U_H(\tau) = [U_{HH}(\tau); U_{VH}(\tau)]$. The total impulse response of this state corresponds to the red plot of Fig 4.a. The numerical propagation of this state using the experimentally

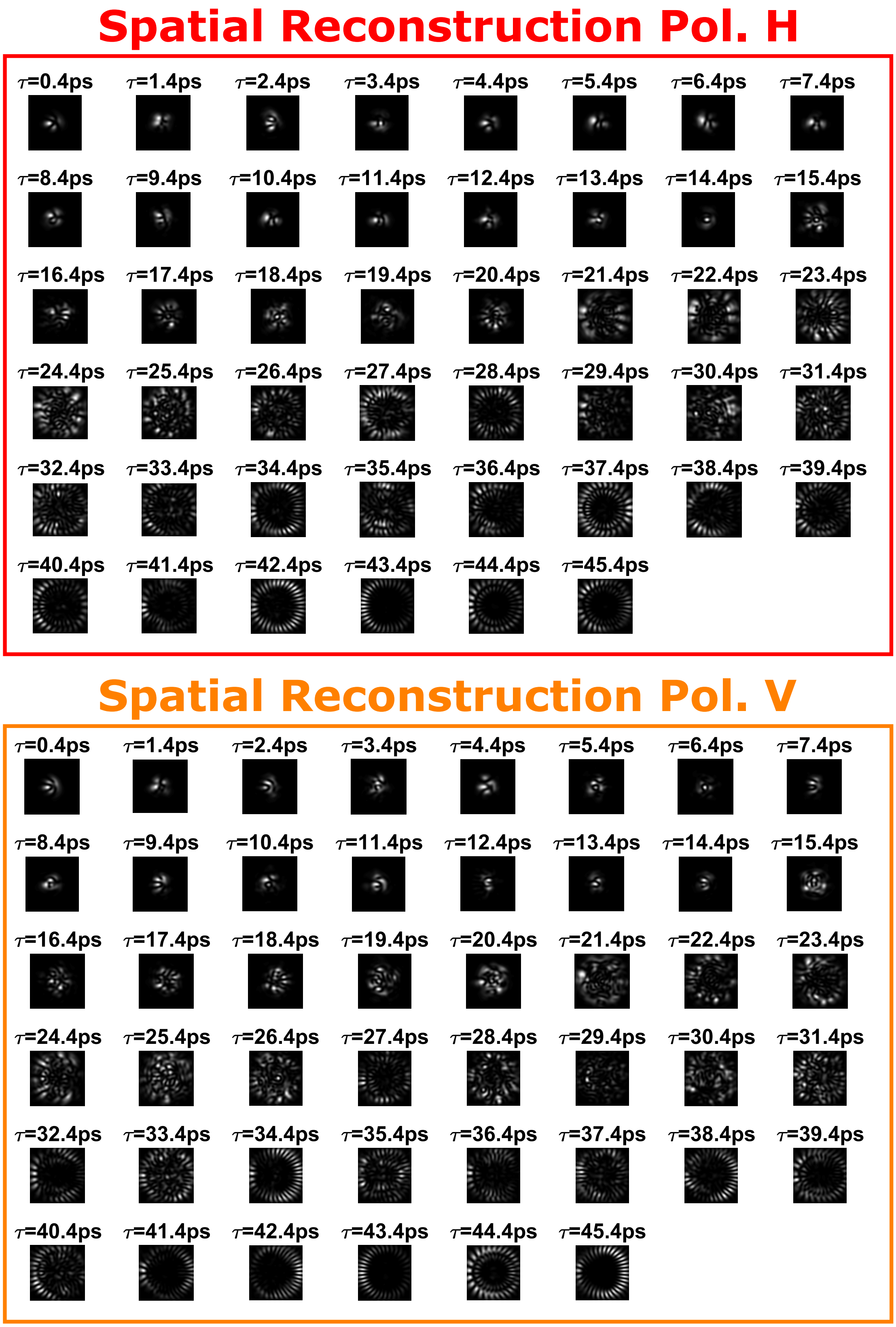

Supplementary Figure 6. Spatial Reconstruction of the max eigenstate intensity images at different delays for both H (left hand side) and V (right hand side). The color-scale is identical for all the plots.

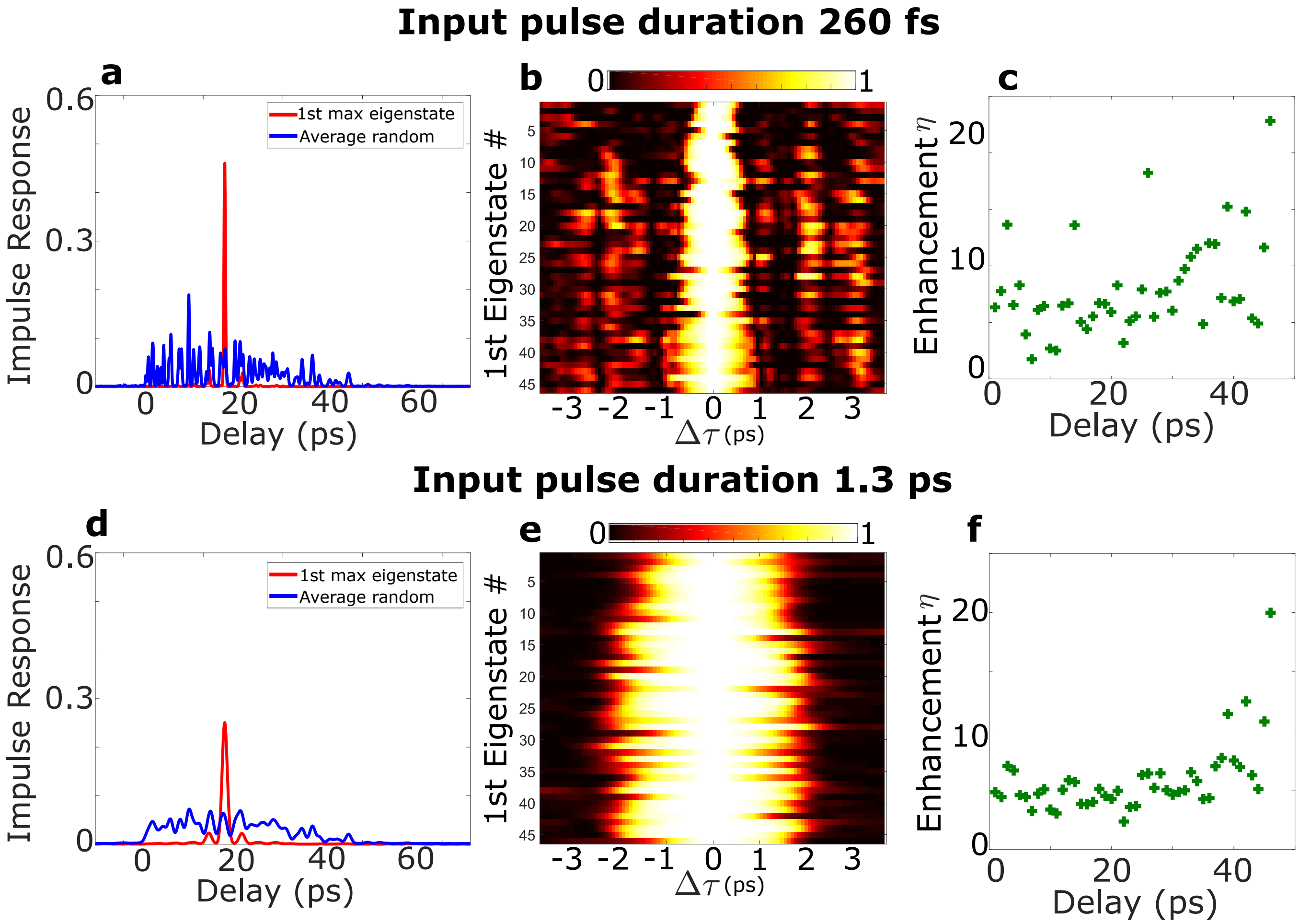

Supplementary Figure 7. Simulation of numerical propagation of an ultrashort pulse of given duration. We show results for two different input pulse durations: (a,b,c) 260fs and (d,e,f) 1.3ps. (a,d) Impulse response when enhancing at τ_{max}=14ps (b,d) square of the field correlation in the temporal domain (c,f) enhancement ratio

measured MSTM is the black plot of Supplementary Figure 8 below. Fig 4.a shows that the peak of the impulse response is almost fully contained onto the H output state (the black plot is the H part of the impulse response, and the orange is the V part). A similar approach can be drawn with the control of the V output state, by replacing $U_H(\tau)$ with $U_V(\tau)$. The numerical propagation of this maximum output V state is the orange plot of Supplementary Figure 8. If now we want to enhance both H and V, we exploit $U(\tau)$ that contains both $U_H(\tau)$ and $U_V(\tau)$. The output power is then split almost evenly between the H output state and the V output state (see the reconstructed field at τ in Fig. 2a). The total impulse response is the red plot of Supplementary Figure 8. The power is similar to the red and orange plot, as we are using both polarization at the input: similar power is then sent in the fiber, and depending on the eigenstate that we launch, we split that power either onto the H output, or the V output or both H and V at the output.

Let's now ignore on purpose one of the polarization at the input. We keep for instance only the H input polarization state. It means that the transmission matrix we can exploit is restricted to $[U_{HH}(\tau); U_{HV}(\tau)]$. We want now to enhance the output H part: we are thus using only $U_{HH}(\tau)$. The total impulse response of this eigenstate is plotted in green in Supplementary Figure 8. We can enhance the H polarization at the output, but we have lost some power because we are only using half the total modes that we could control at the input. The polarization control at the input enables not to waste half of the power if the input beam is polarized.

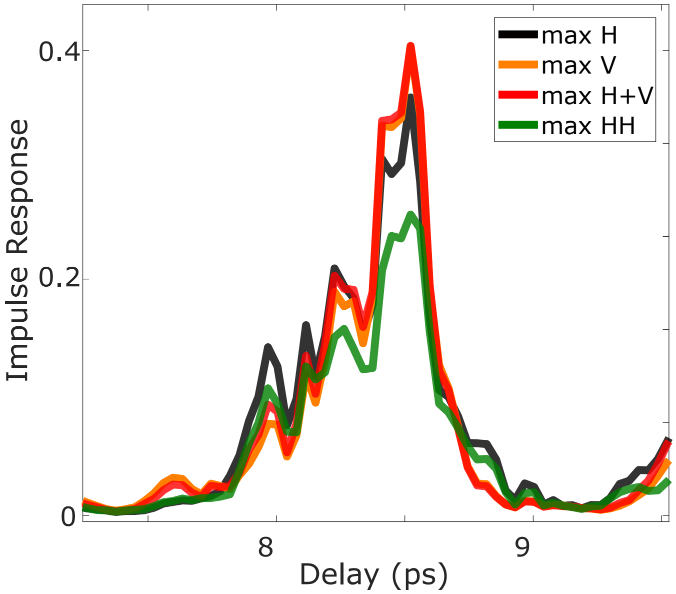

Supplementary Figure 8. Polarization control of the output state depending on the polarization controlled at the input. The impulse responses are calculated with the numerical propagation of a given input eigenstate. They are the combination of the impulse response of the H output and of the V output. We control here light at delay $\tau = 8.5$ ps. (Black plot) Using H and V at the input and maximizing H at the output. (Orange plot) Using H and V at the input and maximizing V at the output. (Red plot) Using H and V at the input and maximizing H and V at the output. (Green plot) Using only H at the input and maximizing H at the output.

Supplementary Note 6. ARBITRARY POLARIZATION STATE AT A CHOSEN DELAY

In order to get a specific polarization state, we need to change the output polarization basis. Similarly to what was shown in the monochromatic regime in [6], we can calculate the time resolved transmission matrix in a different output polarization basis, based on the experimentally measured matrix in the H/V basis. Eq. 4 gives the polarization decomposition of a given time gated transmission matrix U(τ) at delay τ. In principle, the output polarization basis can be numerically rotated at will from U(τ). For instance, we want to access either the left hand or the right hand output polarization states. From these components we can write the matrix $U_{HR}(\tau)$ which is the matrix relating the H input on the SLM to the right hand polarization state at the output: $U_{HR}(\tau) = 1/\sqrt{2}(U_{HH}(\tau) - iU_{HV}(\tau))$. We can calculate also the full matrix on the left hand and right hand circular output basis:

$$\begin{bmatrix} U_{HR}(\tau) & U_{VR}(\tau) \\ U_{HL}(\tau) & U_{VL}(\tau) \end{bmatrix} = 1/\sqrt{2} \begin{bmatrix} 1 & -i \\ 1 & i \end{bmatrix} \begin{bmatrix} U_{HH}(\tau) & U_{VH}(\tau) \\ U_{HV}(\tau) & U_{VV}(\tau) \end{bmatrix} \tag{5}$$

with $U_{ij}(\tau)$ the sub time gated matrix at delay τ connecting the $i-th$ input polarization state to the $j-th$ output polarization state. This process is then equivalent to adding a quarter wave plate at 45 degrees of the horizontal axis onto the output. If we want to enhance the right hand circular polarization state, then we can use the maximum eigenstate of $U_R(\tau) = [U_{HR}(\tau); U_{VR}(\tau)]$. A similar approach can be set with the control of the left hand output state, by replacing $U_R(\tau)$ with $U_L(\tau) = [U_{HL}(\tau); U_{VL}(\tau)]$.

Furthermore, we can also access the linear +45 / -45 degrees from the time resolved transmission matrix in the H/V basis using:

$$\begin{bmatrix} U_{H-45}(\tau) & U_{V-45}(\tau) \\ U_{H+45}(\tau) & U_{V+45}(\tau) \end{bmatrix} = 1/\sqrt{2} \begin{bmatrix} 1 & -1 \\ 1 & 1 \end{bmatrix} \begin{bmatrix} U_{HH}(\tau) & U_{VH}(\tau) \\ U_{HV}(\tau) & U_{VV}(\tau) \end{bmatrix} \tag{6}$$

Note that we could apply a similar method (e.g. change of the output polarization basis) on the multi spectral matrix directly. Instead of measuring H/V on the camera, we could be measuring the left hand circular and the right hand circular polarizations states on both sides of the camera with a quarter wave plate in the experiment, or by applying a numerical quarter wave plate on the output. Or +45 degrees / -45 degrees by applying a rotation matrix on the output. Enhancing only a given polarization state (e.g. either LCP or RCP, or +45 or -45) would then be equivalent to enhancing one side of the camera, as we did for either H or V in Fig. 4 of the manuscript.

We demonstrate in Supplementary Figure 9 how to enhance the total transmission with either left hand polarization or right hand polarization, or linear polarization at either +45 degrees or -45 degrees, at delay τ=15 ps. We use here a numerical propagation, as described in the main manuscript in Section 3, to obtain the polarization and time resolved output fields. We then apply a phase shift φ (16 steps between 0 and 2π) for both H and V to look at the output polarization state for all the different spatial positions. The total intensity is shown on a grey scale color-map that is identical for all the subfigures. The polarization state is represented as a vector, with the size of the arrow related to the amplitude of H and V, and its direction related to the phase difference between H and V. The H axis is set as horizontal, and the V axis as vertical. The top line corresponds to enhancing either H or V at the output, which is demonstrated in Fig. 4 of the main manuscript. The middle line shows the enhanced field with either a left hand or a right hand polarization state, and the bottom line shows both 45 degrees linear polarization states. The expected helicity is observed, as well the expected angle for the linear polarization. Note that only a phase shift has been applied when calculating $U_L(\tau)$ and $U_R(\tau)$, which explains why we don't have a perfect circular state, as the power ratio between H and V differs in all spatial positions.

Supplementary Note 7. MULTIPLE DELAY CONTROL: ADDITIONAL EXAMPLES

In Figure 5, we show multiple delay control by superposing $N_{\text{superpos.}}$ eigenstates at different arrival times. More precisely, we show a superposition of $N_{\text{superpos.}} = 6$ solutions within a 40 ps time interval, and $N_{\text{superpos.}} = 7$ solutions within a 15 ps time interval. We provide additional superposition plots in Supplementary Figure 10 and Supplementary Figure 11, with $N_{\text{superpos.}}$ varying from 2 to 9, within 40 ps in Supplementary Figure 10 and within 15 ps in Supplementary Figure 11.

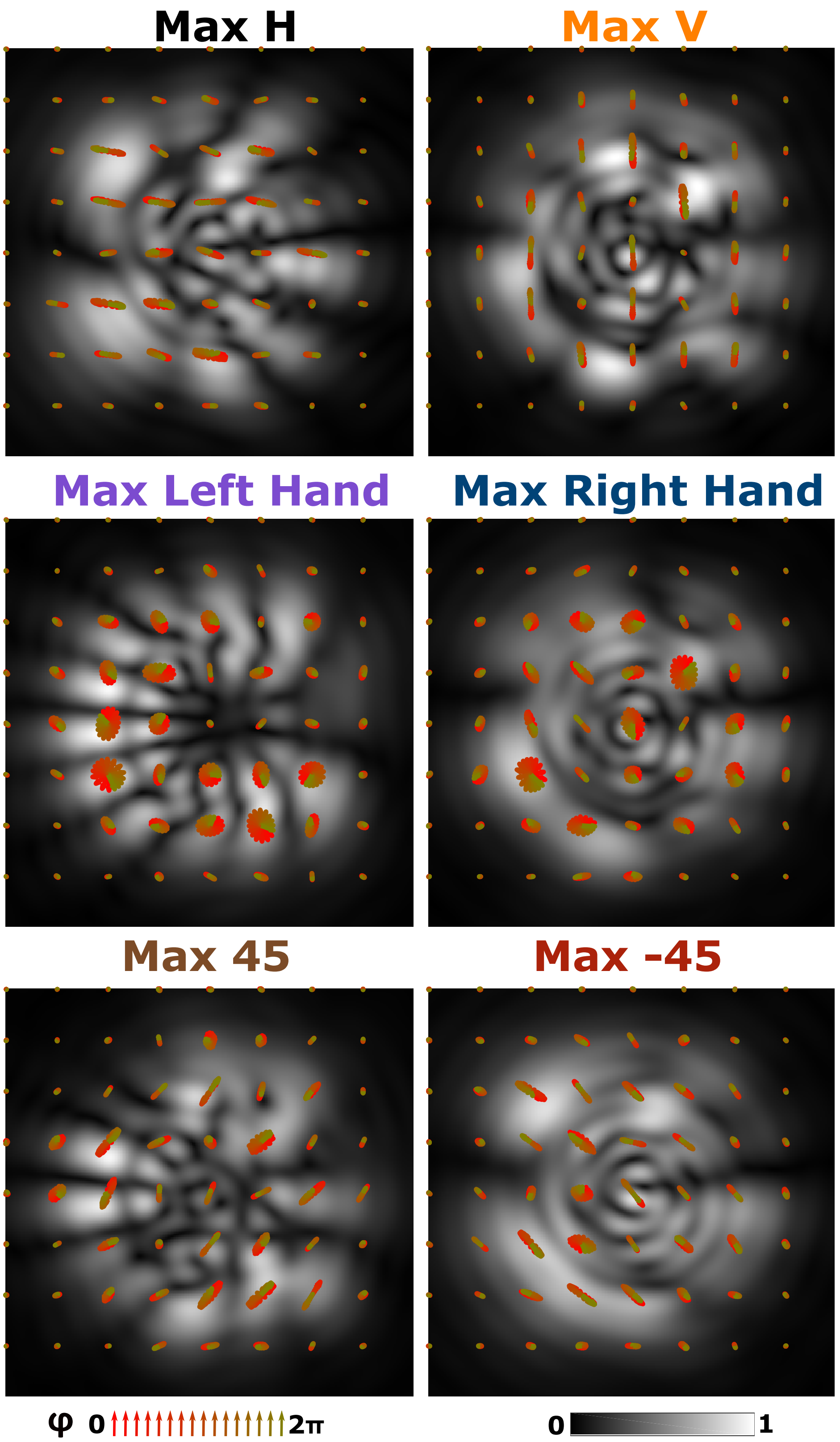

Supplementary Figure 9. (Numerical propagation) Intensity images upon enhancing different output polarization states at τ =15ps with the time resolved transmission matrix. Both the H intensity and the V intensity are superimposed. Same grey level intensity map for all plots. The arrows represent the polarization vector upon a phase shift of 0 (red arrow) to 2π (green arrow) on both H and V components Top left: H; Top right: V; Mid Left: Left hand; Mid right: Right hand; Bottom left: Linear 45 degrees; Bottom right: Linear -45 degrees.

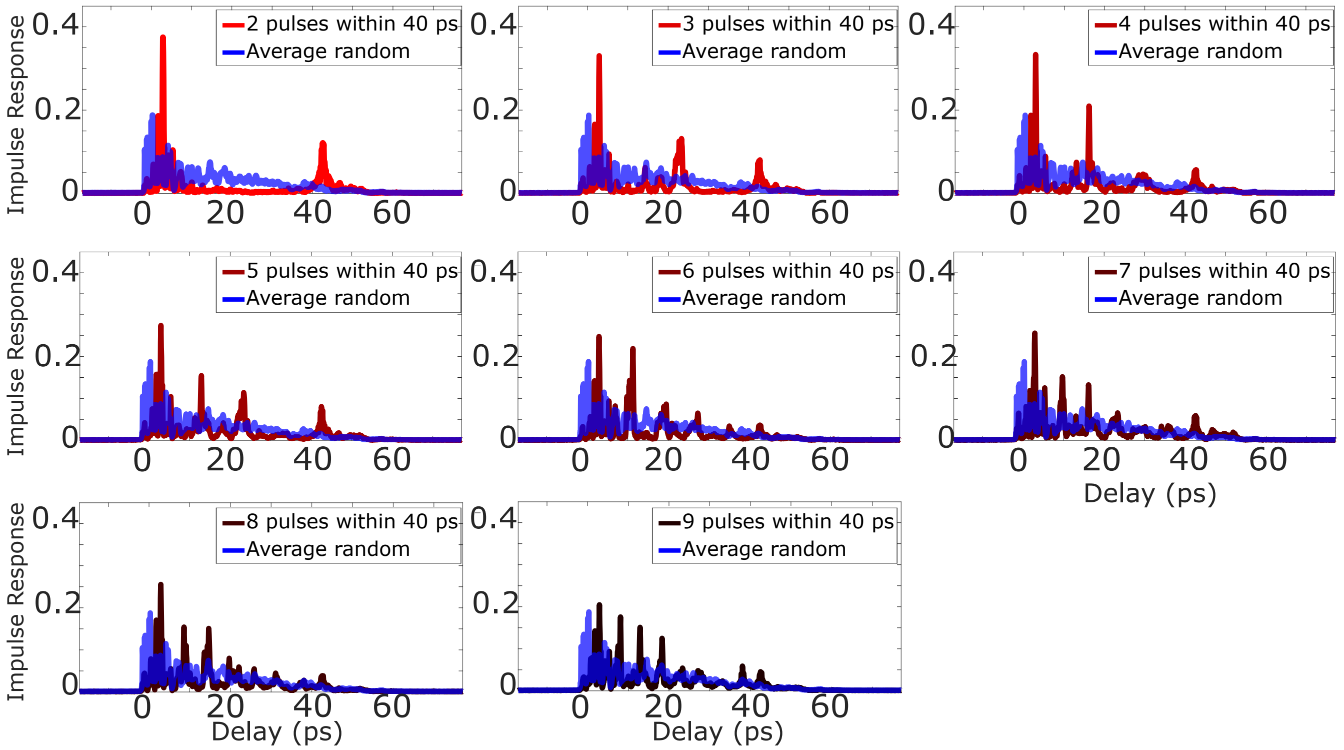

Supplementary Figure 10. Enhancement of transmitted light intensity at $N_{\text{superpos.}}$ multiple delay times between 4 ps and 44 ps, $N_{\text{superpos.}}$ varying from 2 to 9.

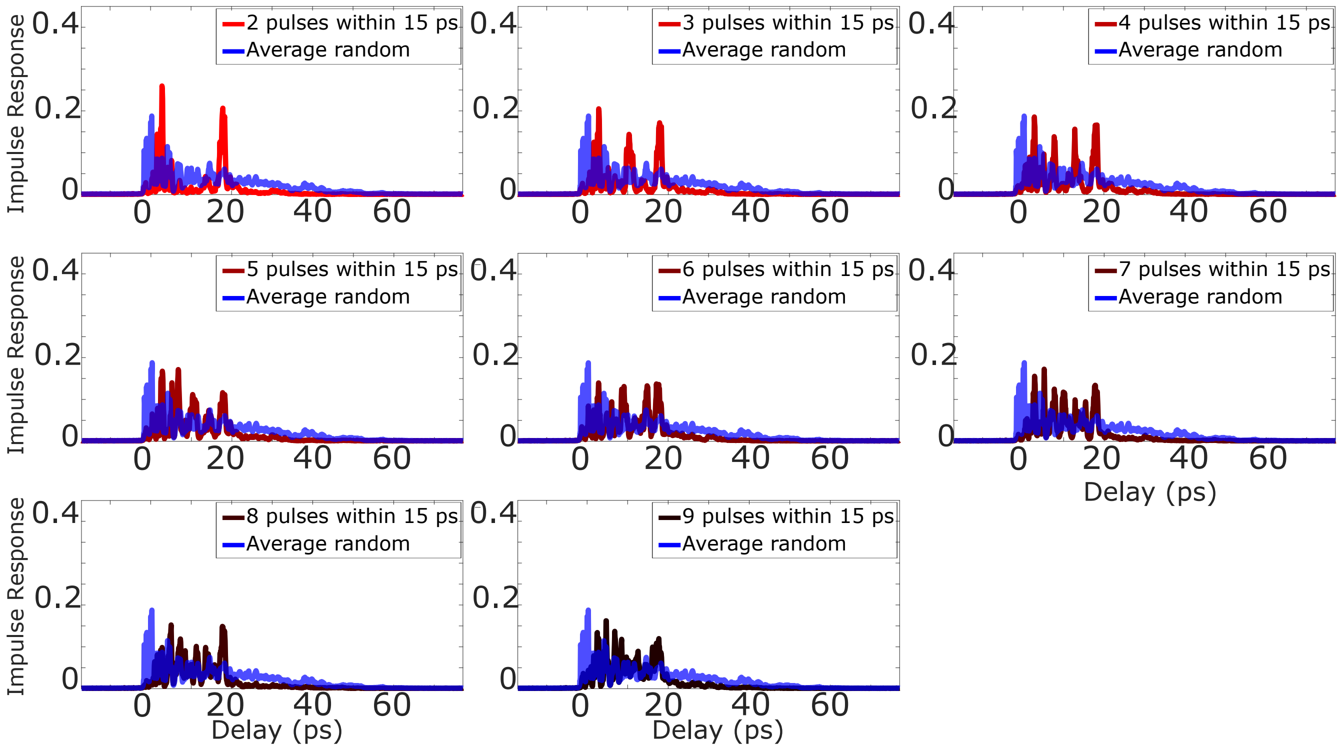

Supplementary Figure 11. Enhancement of transmitted light intensity at $N_{\text{superpos.}}$ multiple delay times between 4 ps and 19 ps, $N_{\text{superpos.}}$ varying from 2 to 9.

Supplementary References

[1] J. Carpenter, B. J. Eggleton, and J. Schröder, Nature Photonics **9**, 751 (2015).
[2] R. W. GERCHBERG, Optik **35**, 237 (1972).
[3] J. Carpenter, B. J. Eggleton, and J. Schröder, Laser & Photonics Reviews , n/a (2016).
[4] W. Xiong, P. Ambichl, Y. Bromberg, B. Redding, S. Rotter, and H. Cao, Optics Express **25**, 2709 (2017).
[5] Z. Shi, M. Davy, J. Wang, and A. Z. Genack, Optics Letters **38**, 2714 (2013).
[6] W. Xiong, C. W. Hsu, Y. Bromberg, J. E. Antonio-Lopez, R. A. Correa, and H. Cao, Light: Science & Applications **7**, 1 (2018).
